# Cell-Wide Survey of Amide-Bonded Lysine Modifications by Using Deacetylase CobB

**DOI:** 10.1186/s12575-019-0109-x

**Published:** 2019-12-01

**Authors:** Yun Wei, Wan-Jie Yang, Qi-Jun Wang, Peng-Cheng Lin, Jian-Yuan Zhao, Wei Xu, Shi-Min Zhao, Xia-Di He

**Affiliations:** 1 Institutes of Biomedical Sciences, Obstetrics & Gynecology Hospital of Fudan University, State Key Lab of Genetic Engineering and School of Life Sciences, Shanghai, People’s Republic of China; 2 NHC Key Lab of Reproduction Regulation (Shanghai Institute of Planned Parenthood Research), Fudan University, Shanghai, 200032 People’s Republic of China; 3 Shanghai Institute of Immunology, Shanghai Jiao Tong University School of Medicine, Shanghai, 200025 People’s Republic of China; 4 Key Laboratory for Tibet Plateau Phytochemistry of Qinghai Province, College of Pharmacy, Qinghai University for Nationalities, Xining, 810007 People’s Republic of China

**Keywords:** Lysine post-translational modification, CobB deacetylase, Proteome

## Abstract

**Background:**

Lysine post-translational modifications are important regulators of protein function. Proteomic and biochemical approaches have resulted in identification of several lysine modifications, including acetylation, crotonylation, and succinylation. Here, we developed an approach for surveying amide-bonded lysine modifications in the proteome of human tissues/cells based on the observation that many lysine modifications are amide-bonded and that the *Salmonella enterica* deacetylase, CobB, is an amidase.

**Results:**

After the proteome of human tissues/cells was denatured and the non-covalently bonded metabolites were removed by acetone washes, and the amide-bonded modifiers were released by CobB and analyzed using liquid- and/or gas chromatography/mass spectrometry metabolomic analysis. This protocol, which required 3–4 days for completion, was used to qualitatively identify more than 40 documented and unreported lysine modifications from the human proteome and to quantitatively analyze dynamic changes in targeted amide-bonded lysine modifications.

**Conclusions:**

We developed a method that was capable of monitoring and quantifying amide-bonded lysine modifications in cells of different origins.

## Background

Many amino acids are modified post translationally to regulate functions of proteins. Adding phosphor groups to serine, threonine and tyrosine constitutes protein phosphorylation and one of the major mechanisms of cellular signal transduction [1, 2]. Other amino acid residues such as histidine, proline and cysteine can also be phosphorylated [3, 4], hydroxylated [5] or acylated [6], respectively, to convey various biological functions. Lysine is the most heavily modified residue in proteins. More than 90 different lysine modifications have been identified on lysine [7]. Many lysine modifications are physiologically significant and have been studied extensively. For example, lysine acetylation of histones and other nuclear proteins is critical for chromatin remodeling and regulation of gene transcription [8, 9], whereas acetylation of metabolism-related enzymes is important for regulation of metabolism [10, 11]. Lysine methylation marks histones and other proteins for regulation of transcription and protein activity [12]. Although lysine methylation is an important modification, the majority of lysine modifications are amide-bonded. This was recently confirmed after an array of metabolites, including propionate, butyrate, malonate, crotonate, and succinate and their respective coenzyme A derivatives, was shown to contain amide-bonded lysine residues [13–16].

Cell-wide survey of known lysine modifications, such as acetylation [17] or methylation [18], has been documented. However, a more comprehensive understanding of the importance of lysine modifications will require elucidation of the cell-wide dynamics and types of lysine modifications. Thus, protocols for systematically and quantitatively surveying different types of lysine modifications are necessary. Because most lysine modifications are formed via amide bonds, cell-wide surveys of lysine modifications can be conducted once a nonspecific amidase is identified.

Accordingly, in this study, we developed an approach for surveying amide-bonded lysine modifications in the proteome of human tissues/cells.

## Results

**Fig. 1:**
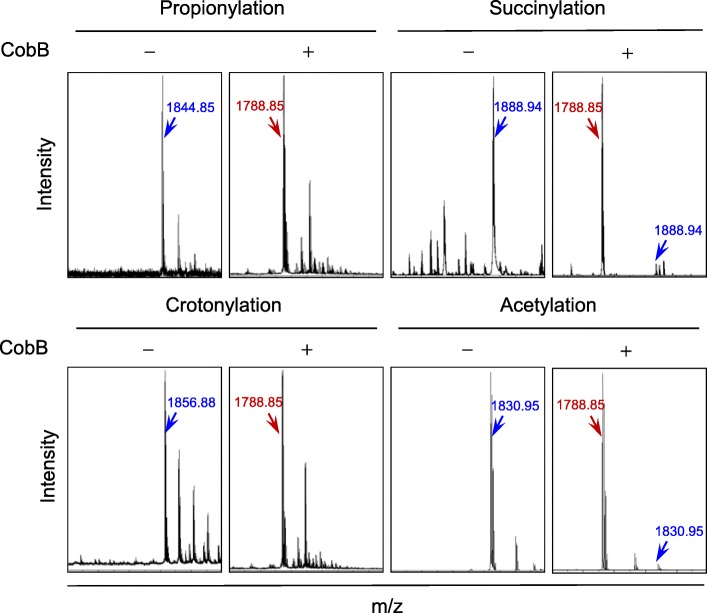
CobB is an amidase. The abilities of CobB to cleave synthetic propionylated, succinylated, crotonylated and acetylated peptides were tested. The M/Z values of synthetic (left each group) and cleaved (right each group) peptides were determined by mass spectrometry and marked

**Fig. 2:**
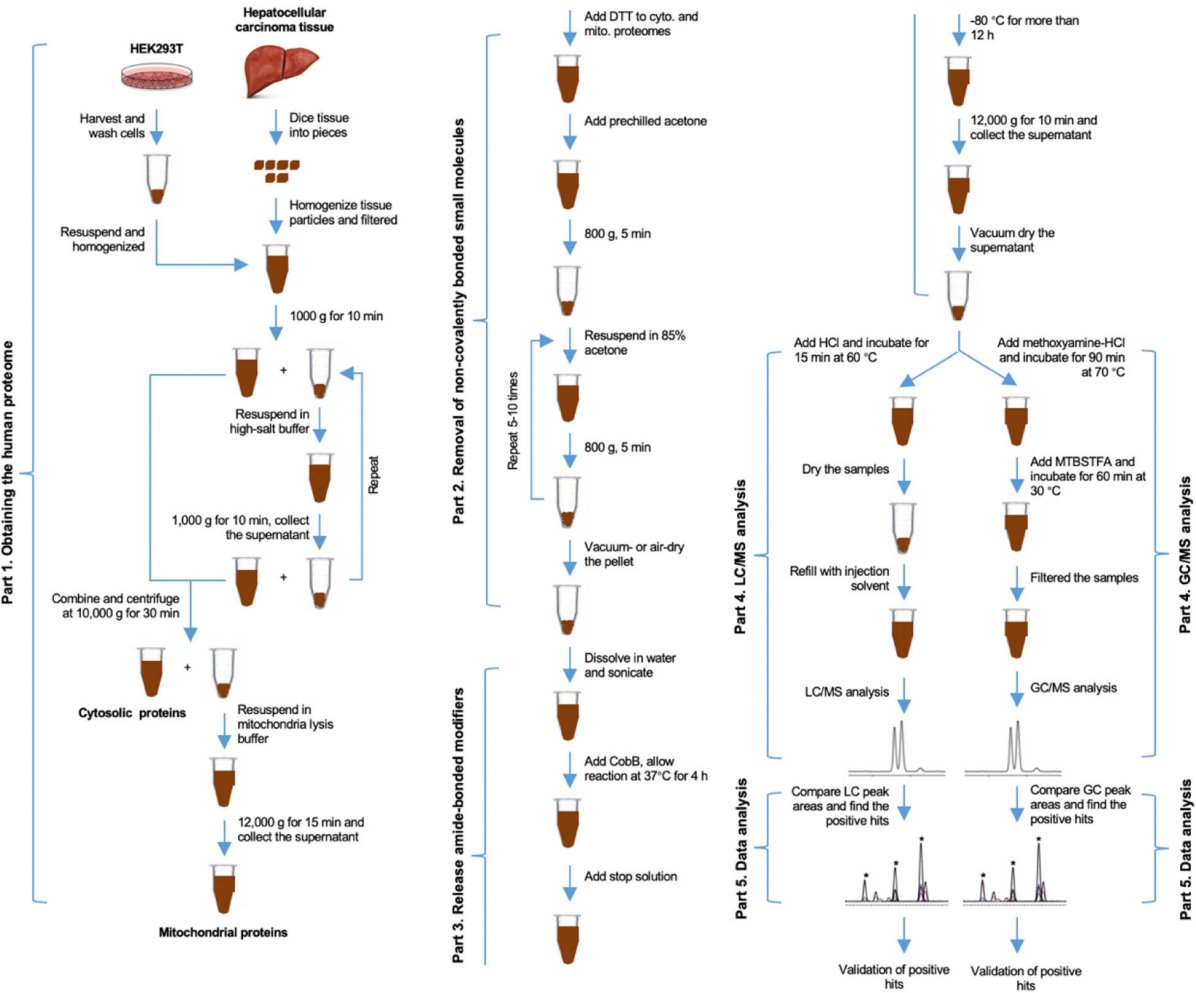
Flow chat of cell-wide survey for amide-bonded lysine modifications. Lysates were obtained and depleted for non-covalently bound metabolites, amide-bonded modifiers were released with CobB and detected with GC/UPLC-MS

While investigating the activities of *Salmonella enterica* deacetylase CobB [10], we unexpectedly observed that CobB possesses nonspecific amidase activity. Recombinant CobB purified from *Escherichia coli* efficiently cleaved all tested amide-bonded lysine modifications, including propionylation, succinylation, crotonylation, and acetylation, on synthetic peptides (Fig. 1). This suggested that CobB could be used as a general amidase to release amide-bonded lysine modifiers and thereby identify novel lysine modifications in cells. Thus, we developed a CobB-based protocol to survey amide-bonded lysine modifications in human cells. Briefly, proteins in cell lysates were precipitated and washed extensively with acetone to remove any small molecules that were noncovalently bound to the proteins, the amide-bonded modifications to the proteome were released by CobB treatment, and the released modifiers were analyzed by liquid chromatography/mass spectrometry (LC/MS) or gas chromatography/mass spectrometry (GC/MS). Metabolites, the levels of which were significantly higher in CobB-treated samples than in untreated control samples, were considered possible lysine modifiers (Fig. 2) because only the ε-amine of lysine and the N-terminus amine of a protein could form amide bonds; however, we confirmed that the amide bonds formed with the N-terminal amine were not cleaved by CobB. Using this protocol, we successfully identified more than 40 new lysine modifications, including lysine aminoacylations, which facilitate sensing and signal transduction of intracellular amino acids in human liver cancer tissues and HEK293T cells [19].

**Fig. 3:**
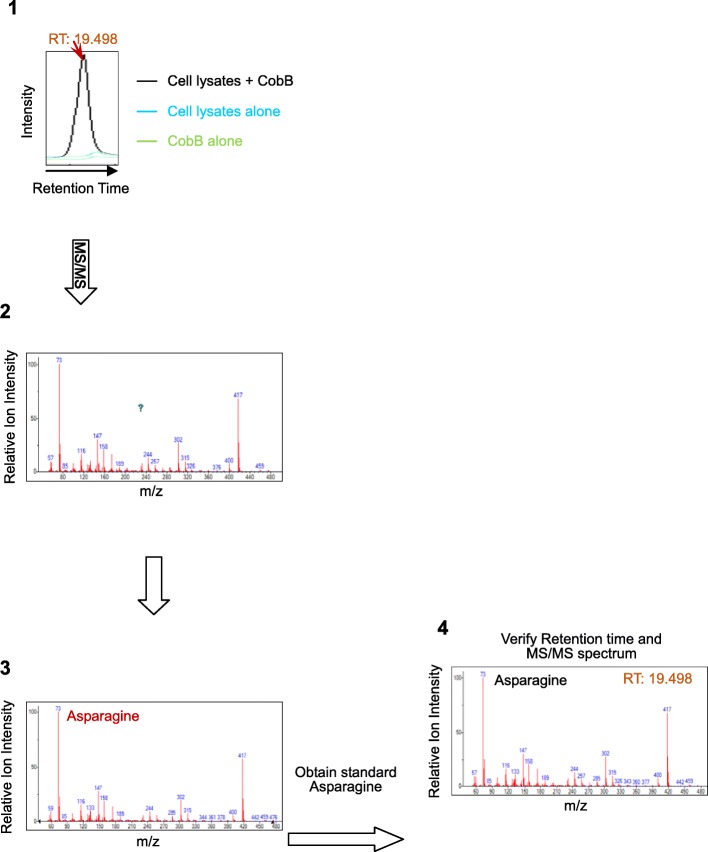
An example flow chat of how a positive identification (asparagine) was obtained. A GC peak at certain retention time (19.498 min) is generated by CobB treatment (**1**), the MS/MS spectrum of the 19.498 min peak generated by CobB treatment (**2**), Searching against NIST library identified that the metabolite of 19.498 min peak was asparagine (**3**), the standard asparagine generate the same GC retention time and MS/MS spectrum as the metabolite generated by CobB treatment (**4**)

A successful survey using the above described protocol will generate the following results: 1) significantly higher (> 2-fold, *p* value < 0.05) GC or LC peak areas should be obtained in CobB-treated samples than in matched untreated samples (Fig. 3-1); 2) the MS/MS fingerprint spectra (Fig. 3-2) should match some of the spectra in the NIST mass spectral library (Fig. 3-3); 3) the retention time in GC or LC and the MS/MS spectrum of identified metabolites should match those of authentic standard metabolites (Fig. 3-4). Insignificant increases (< 2 fold, *p* value < 0.05) in GC or LC peak areas are often related to insufficient removal of non-covalently bonded metabolites or CobB inactivation; a lack of GC or LC peaks with significant increases in area may be related to the first reason, whereas a lack of significant increases in GC or LC peak areas may be related to the second reason. The protocol can be improved accordingly.

## Discussion

With the increasing discovery of the importance of PTMs, there is a growing need for discovery of novel PTMs. This protocol represents a general method for identifying and quantitatively analyzing amide-bonded modifications in cells. By using this method, it is not difficult to find dozens of new PTMs. Although we successfully used the method to identify amide-bonded lysine modifications in human kidney and liver cancer cells [19], this approach could also be applied to cell types of other human tissues or origins, as long as the proteome of these cells (bacteria, plants, and other mammalian cells) is readily available. Moreover, a well-designed and well-performed analysis will allow quantification of the dynamic changes in cellular amide-bonded lysine modifications. Of note, the principle of our survey can be expanded to detect other PTMs of distinct chemical natures. For example, if a proper cleaving enzyme is identified, modifications such as acylation in cysteine, ubiquitinoylation or SUMOylation in glycine and glycosylation on various amino acids can be screened cell wide.

Lysine modifications are identified using distinct approaches (Table 1). For example, investigations on biochemical reactions have led to the identification of lysine acetylation [20] and methylation [21], and proteomic analysis has resulted in the identification of a number of lysine modifications, including propionylation [13], butyrylation [13], and crotonylation [16]. Our current protocol utilized the amidase activity of CobB in combination with metabolomic analysis to identify multiple lysine modifications. To the best of our knowledge, this is the first protocol to allow cell-wide identification and quantification of amide-bonded lysine modifications.

**Table 1:** Comparison with other methods

	Advantage	Disadvantage
Our method	1. Multiple modifications identification 2. Known the demodification enzyme	1. Only amide-bond lysine modifications 2. Higher false positive ratio 3. More expertise needed
*Biochemical* *Journal*, 1963	1. Lower false positive ratio 2. Less expertise needed	1. Only amide-bond lysine modifications 2. One modification identification 3. Unkown the demodification enzyme
*Molecular & Cellular Proteomics, 2007*	1. Lower false positive ratio 2. Less expertise needed	1. Only amide-bond lysinemodifications 2. One modification identification 3. Unkown the demodification enzyme

We confirmed that CobB did not cleave the N-terminus amine-derived amide bonds and peptide bonds in the protein backbone, suggesting that modifiers obtained after CobB cleavage were putative lysine modifiers [19]. However, the protocol had two limitations. First, there was a slight possibility that the CobB-cleaved modifiers were not lysine modifiers because we did not test whether CobB could cleave other chemical bonds. Second, although we identified an array of new lysine modifications using this protocol [19], whether this approach may enable complete identification of amide-bonded lysine modifications was unclear because the ability of CobB to cleave all types of amide-bonded modifications could not be exhaustively tested. Nevertheless, our protocol represents a useful tool for surveying potential novel lysine modifications.

## Conclusions

This method was developed because of the lack of method for systematically and quantitatively surveying different types of lysine modifications. We propose a method that can be accurate, facile, and reproducible to identify and quantify new amide-bonded lysine modifications in cells of different origins.

## Methods

### Reagents

Methanol (Sigma, St. Louis, MO, USA; cat. no. 34860-1 L-R).

**! CAUTION** Methanol is highly flammable and toxic, handle with caution.

Pyridine (Sigma; cat. no. 270407-1 L).

Methoxamine hydrochloride (Sigma; cat. no. 226904).

N-tert-Butyldimethylsilyl-N-methyltrifluoro-acetamide (MTBSTFA; Sigma; cat. no. 394882).

Tris base (Sangon, cat. no. TB0194).

Tryptone (OXOID; cat. no. LP0042).

Yeast extracts (OXOID; cat. no. LP0021).

NaCl (Sangon; cat. no. SB0476).

Isopropyl β-d-1-thiogalactopyranoside (IPTG; Sangon; cat. no. A100487).

Dithiothreitol (DTT; Merck; cat. no. 233156).

Adonitol (Sigma; cat. no. A5502).

Ampicillin (Sangon; cat. no. A610028).

Urea (Sangon; cat. no. A510907).

Imidazole (Sangon; cat. no. A500529).

Mitochondrial lysis buffer (Pierce; cat. no. 89874).

Sodium dodecyl sulfate sodium salt (SDS; Sangon; cat. no. 0227).

Ammonium persulfate (Sigma; cat. no. A3678).

Acrylamide (Sangon; cat. no. 0341).

Bisacrylamide (Sangon; cat. no. 0172.

**! CAUTION** Acrylamide and bisacrylamide are potent neurotoxins. Use appropriate safety measures, such as protective gloves and safety goggles, and handle under adequate ventilation.

N",N",N",N"-tetramethylenethylendiamide (BI; cat. no. TB0508).

β-Mercaptoethanol (AMRESCO; cat. no. 0482).

**! CAUTION** β-Mercaptoethanol fumes are toxic and should be dispersed under a fume hood.

Nicotinamide (Sigma; cat. no. N0636).

Aprotinin (BBI; cat. no. AD0153).

Leupeptin (Sangon; cat. no. LJ580).

Pepstain (Sangon; cat. no. J583).

KCl (BBI; cat. no. PB0440).

Nicotinamide adenine dinucleotide (Sigma; cat. no. N8285).

Mitochondrial lysis buffer (Pierce; cat. no. 89874).

HEPES (Sangon; cat. no. H0511).

Ethylenediaminetetraacetic acid (EDTA; Sangon; cat. no. 0105).

MgCl_2_ (Fluka; cat. no. 63063).

1-Butanol (Sigma; cat. no. B7906).

HyClone phosphate-buffered saline (PBS), 10× (GE Healthcare; cat. no. SH30258.01).

Acetone (Sigma; cat. no. 270725).

**! CAUTION** Acetone can damage the mucosa of the mouth and can irritate and damage skin. Acetone should be used with appropriate safety measures, such as protective gloves, glasses, and clothing as well as adequate ventilation. Acetone should be stored in an explosion- or flame-proof freezer.

Coomassie brilliant blue G-250 (Serva; cat. no. 17524).

Protein Assay Dye Reagent Concentrate (for Bradford Assay; Bio-Rad, Hercules, CA, USA; cat. no. 500–0006).

**! CAUTION** This reagent is toxic; handle with care.

Hydrochloric acid (HCl; EMD Millipore; cat. no. HX0603–4).

**! CAUTION** HCl is highly corrosive and toxic; handle with care.

Alanine (Sigma; cat. no. 05129).

Aspartate (Sigma; cat. no. A9310–0).

Asparagine (Sigma; cat. no. A0884).

Arginine (Sigma; cat. no. A5006).

Cysteine (Sigma; cat. no. V900400).

Glycine (Sigma; cat. no. 4102250).

Glutamine (Sigma; cat. no. G3126).

Glutamate (Sigma; cat. no. G8415).

Histidine (Sigma; cat. no. H6034).

Isoleucine (Sigma; cat. no. I2752).

Lysine (Sigma; cat. no. 62840).

Leucine (Sigma; cat. no. L8912).

Methionine (Sigma; cat. no. V900487).

Phenylalanine (Sigma; cat. no. V900615).

Proline (Sigma; cat. no. P0380).

Serine (Sigma; cat. no. S4311).

Tyrosine (Sigma; cat. no. T3754).

Threonine (Sigma; cat. no. T8625).

Tryptophan (Sigma; cat. no. V900470).

Valine (Sigma; cat. no. V0500).

**! CAUTION** All experiments should be performed in accordance with relevant guidelines and regulations. A physician or a nurse practitioner must obtain informed consent from donors.

### Equipment

0.45-μm Filter-Top Bottle (Corning, Corning, NY, USA; Cat. No. 430625)

100-μm-diameter cell filter (Thermo; cat. no. 08–771-19).

50-mL conical tubes (Thermo; cat. no. 339652).

15-mL conical tubes (Thermo; cat. no. 339650).

1.5-mL Tubes (Axygen; Cat. No. MCT-150-C)

Dounce homogenizer (Active Motif; cat. no. 40401).

Concentrator 5301 (Eppendorf; cat. no. 5301).

Centrifuge 5810R (Eppendorf; cat. no. 5810R).

Centrifuge 5424R (Eppendorf; cat. no. 5424R).

Vacuum drier (Eppendorf; cat. no. Concentrator plus).

Gel electrophoresis apparatus (Bio-Rad; cat. no. 1658001).

Power supply (500 V, 500 mA; SDS-polyacrylamide gel electrophoresis, PAGE; Bio-Rad; cat. no. 1645050).

Typhoon Trio imaging system (GE Healthcare; cat. no. FLA9500).

Fast protein liquid chromatography (FPLC) system with pumps, UV detector, fraction collector, and 2-mL sample loop (GE Healthcare; cat. no. AKTA-FPLC).

Nickel resin column (GE Healthcare; cat. no. 17–5217-01).

Desalination column (GE Healthcare; cat. no. 17–1408-01).

9-mm tread screw neck vial (ANPEL; cat. no. VAAP-32009E-1232-100).

0.22-μm Syringe-Driven Filter Unit (Millex; Cat. No. SLGV004SL)

Low-temperature ultra-high pressure continuous flow cell disrupter (JNBIO; cat. no. JN-3000 PLUS).

Gas chromatograph (Agilent; 7890B gas chromatograph).

Mass spectrometer (Agilent; 5977B mass spectrometer).

− 80 °C freezer (Thermo; cat. no. Forma 700).

Termovap Sample Concentrator (Organomation; cat. no. KL-11250-JG).

### Reagent Setup

#### Homogenization Buffer

Homogenization buffer was composed of 10 mM KCl, 1.5 mM MgCl_2_, 10 mM Tris, and 5 mM nicotinamide. The solution was prepared by dissolving 0.373 g KCl, 0.071 g MgCl_2_, 0.606 g Tris, and 0.305 g nicotinamide in 400 mL deionized water, adjusting the pH to 7.5 with HCl, and adding deionized water to a final volume of 500 mL. This solution can be stored for up to 1 month at room temperature before use.

#### Oximation Mix (for GC/MS)

This solution was composed of 250 mM methoxyamine hydrochloride in pyridine. This solution was prepared by dissolving 20 mg methoxyamine hydrochloride in 1 mL pyridine. This solution must be freshly prepared on the day of the experiment.

#### Derivatization Mix (for GC/MS)

This solution was composed of 20% (v/v) MTBSTFA in pyridine. This solution was prepared made by dissolving 200 μL MTBSTFA in 800 μL pyridine. This solution must be freshly prepared on the day of the experiment.

#### Derivatization Mix (for LC/MS)

This solution was composed of 3 M HCl in 1-butanol. This solution was prepared by dissolving 250 μL HCl in 750 μL 1-butanol. This solution must be freshly prepared on the day of the experiment.

#### High-Salt Buffer

This solution was composed of 20 mM Tris, 25% (v/v) glycerol, 1.5 mM MgCl_2_, 0.2 mM EDTA, and 1.2 M KCl. The solution was prepared by dissolving 1.211 g Tris, 12.5 mL glycerol, 0.071 g MgCl_2_, 0.029 g EDTA, and 44.730 g KCl in 400 mL deionized water; adjusting the pH to 7.4 with HCl; and adding deionized water to a final volume of 500 mL. This solution can be stored for up to 1 month at room temperature before use. Add protease inhibitors described below to a 1× concentration just before use for cell lysis.

#### Coomassie Brilliant Blue

This solution was prepared with 0.25% (w/v) Coomassie brilliant blue G-250, 45% (v/v) methanol, and 10% (v/v) acetic acid. G-250 brilliant blue powder was solubilized in methanol, acetic acid was added, and deionized water was added to an appropriate volume. The solution was filtered through a 0.45-μm filter-top bottle before immediate use and could be stored at room temperature for future use.

#### Acrylamide:Bisacrylamide Solution (for Making SDS Gels)

This solution consisted of 250 g acrylamide dissolved in 417 mL deionized water. The solution was prepared by stirring the acrylamide solution overnight before adding 1.67 g bisacrylamide. The solution was filtered through a 0.45-μm filter-top bottle before use and could be stored at room temperature (20–25 °C) in a dark container.

#### Electrophoresis Running Buffer

The buffer was prepared by dissolving 3.02 g Tris base, 18.8 g glycine, and 1 g SDS in 1 L deionized water. This buffer could be stored at room temperature until use.

#### PBS

PBS was composed of 137 mM NaCl, 8 mM Na_2_HPO_4_, 2.7 mM KCl, and 1.47 mM KH_2_PO_4_, pH 7.1, in deionized water. The solution was prepared by dissolving 8 g NaCl, 1.14 g Na_2_HPO_4_, 200 mg KCl, and 200 mg KH_2_PO_4_ in 800 mL deionized water; adjusting the pH to 7.1 with HCl; and bringing the volume up to 1 L with additional deionized water. The solution was autoclaved in a glass bottle to sterilize. This buffer can be stored at room temperature for at least several months.

#### Binding Buffer (for CobB Purification)

Binding buffer was composed of 100 mM sodium phosphate, 150 mM NaCl, and 20 mM imidazole. The solution was prepared by dissolving 8.197 g sodium phosphate, 4.383 g NaCl, and 0.681 g imidazole in 400 mL deionized water; adjusting the pH to 7.2 with HCl; and adding deionized water to a final volume of 500 mL. This solution must be freshly prepared on the day of the experiment.

#### Elution Buffer (for CobB Purification)

Elution buffer was composed of 100 mM sodium phosphate, 150 mM NaCl, and 300 mM imidazole. The solution was prepared by dissolving 8.197 g sodium phosphate, 4.383 g NaCl, and 10.212 g imidazole in 400 mL deionized water; adjusting the pH to 7.2 with HCl; and adding deionized water to a final volume of 500 mL. This solution must be freshly prepared on the day of the experiment.

### Desalination Buffer (for CobB Purification)

Desalination buffer was composed of 50 mM Tris and 150 mM NaCl. The solution was prepared by dissolving 3.029 g Tris base and 4.383 g NaCl in 400 mL deionized water, adjusting the pH to 7.5 with HCl, and adding deionized water to a final volume of 500 mL. This solution can be stored for up to 1 month at room temperature before use.

#### Protease Inhibitor Cocktail (50×)

This solution contained 50 mM PMSF, 0.05 mg/mL aprotinnin, 0.05 mg/mL leupeptin, and 0.05 mg/mL pepstatin in dimethyl sulfoxide. This solution can be stored for up to 12 weeks at − 20 °C and diluted 50-fold (v/v) before use.

▲**CRITICAL** 1 mM DTT and PMSF should be added to the diluted protease inhibitor cocktail solution before use.

**! CAUTION** Protease Inhibitor Cocktail is extremely toxic; handle with care.

#### Stop Solution (for CobB Cleavage)

Prechilled (− 80 °C) methanol.

### Experimental Design

In addition to the cleavage of amide-bonded lysine modifiers by CobB, the key to the success of this protocol was related to obtaining precleaned proteomes. To achieve this goal, the proteomes of human cells were denatured using 85% acetone, which allowed further removal of non-covalently bonded metabolites while preserving covalently bonded lysine modifications and inactivated enzymes that may remove the modification. The denatured proteome was subject to extensive washing with 85% acetone to remove non-covalently bonded metabolites. The “precleaned” proteome was then subjected to CobB treatment to release the amide-bonded lysine modifiers, and the cleaved modifiers were obtained by collecting the supernatants from the centrifuged denatured CobB reaction mixture. The cleaved modifiers in the supernatant were directly assayed using LC/MS, GC/MS, or both after the modifiers were oximated by methoxyamine hydrochloride. Notably, CobB-untreated samples should be run in parallel with CobB-treated samples as controls.

### Procedure

This protocol can be used to survey lysine modifications (steps **1–59,** Fig. 2) or to quantify targeted lysine modifications (Table 2).

**Table 2:** Quantification of targeted lysine modifications TIMING: 3–4 days for each run

1. Synthesize peptides containing targeted lysine modifications as internal calibrators.	
▲CRITICAL The purity of the synthetic peptides should be greater than 98% to ensure accurate quantification.	
2. Mix known concentrations of synthetic peptide into samples to be analyzed.	
3. Run samples with known synthetic peptides and samples without synthetic peptides through steps 1–54.	
4. Compare the areas of GC or LC peaks of the targeted modifiers in these samples.	
5. Calculate the levels of modifications in these samples by comparing with the internal peptide calibrator.	
6. Alternatively, obtain a working curve by running various levels of synthetic peptides through steps 1–54, and compare areas of GC or LC peaks of samples with the working curve.	

#### Obtaining the Human Proteome ●TIMING: 1–5 H

1. The human proteome was obtained from either hepatocellular carcinoma (HCC) tissue or HEK293T cultured human embryonic kidney cells. HCC tissues were obtained with consent from the patient and processed (start from step 2) within 2 h of the patient’s operation. HEK293T cells were processed (start from step 13) within 15 min of harvesting.
  ▲**CRITICAL** The following steps (**2–22**) must be performed on ice.
2. Dice 2 g HCC tissue into pieces of approximately 0.1 mm using a razor.
3. Resuspend the tissue particles in 10 mL ice-cold (4 °C) homogenization buffer supplemented with 1× protease inhibitor cocktail.
4. Homogenize tissue particles in a Dounce homogenizer with at least 40 strokes.
5. Pass the homogenized suspension through a 100-mm-diameter cell filter (pore size 125 μM) to remove debris.
6. Centrifuge the filtered solution at 1000×*g* for 10 min at 4 °C, and collect the supernatant.
7. Resuspend the pellet from **step 6** in 10 mL ice-cold (4 °C) homogenization buffer supplemented with 1× protease inhibitor cocktail by vortexing.
8. Recentrifuged the samples at 1000×*g* for 10 min at 4 °C, and collect the supernatant.
9. Resuspend the pellet from **step 8** in 2 mL high-salt buffer with vortexing, and place the suspension on a rotary shaker for 30 min at 4 °C to extract nucleic proteins.
10. Centrifuge at 1000×*g* for 10 min at 4 °C, and collect the supernatant.
11. Repeat **steps 9–10**, and collect the supernatant.
12. Combine the supernatants from **steps 6, 8, 10, and 11**, and go to **step 23**.
13. Harvest two dishes (15 cm) of confluent HEK293T cells by scraping, and wash the cells three times using 10 mL PBS with centrifugation at 1000×*g*.
14. Resuspend the cells in 10 mL ice-cold (4 °C) homogenization buffer supplemented with 1× protease inhibitor cocktail.
15. Homogenize cells in a Dounce homogenizer with at least 40 strokes.
16. Centrifuge the homogenized mixture at 1000×g for 10 min at 4 °C, and collect the supernatant.
17. Resuspend the pellet from **step 14** by vortexing in 10 mL ice-cold (4 °C) homogenization buffer supplemented with protease inhibitor cocktail.
18. Centrifuge the samples at 1000×*g* for 10 min at 4 °C, and collect the supernatant.
19. Repeat **steps 15–16**, and collect the supernatant.
20. Resuspend the pellet from **step 17** in 2 mL high-salt buffer with vortexing to extract nuclear proteins, and place the suspension on a rotary shaker for 30 min at 4 °C.
21. Centrifuge at 1000×*g* for 10 min at 4 °C, and collect the supernatant.
22. Combine the supernatants from **steps 14, 16, 17, and 19**, and go to **step 23**.
23. Centrifuge the supernatant at 10,000×*g* for 30 min. Separate the supernatant (which contains cytosolic and nuclear proteins; for further processing, go to **step 26**) and pellet (which contains mitochondria; for further processing, go to **step 24**) by carefully removing the supernatant using a pipette. Keep both fractions for further treatments.
24. Resuspend the pellet from **step 23** in 1 mL mitochondria lysis buffer supplemented with protease inhibitor cocktail, and rotate gently at 4 °C for 30 min on a rotary shaker.
25. Centrifuge at 12,000×*g* for 15 min at 4 °C, collect the supernatant (which contains mitochondrial proteins), and discard the pellet.

#### Removal of Non-covalently Bonded Small Molecules from the Denatured Human Proteome ●TIMING 2–3 H

26. Add DTT (final concentration: 1 mM) to the cytosolic (from **step 23**) and mitochondrial (from **step 25**) proteomes to break disulfide bonds within proteins. Allow the reaction to proceed for 10 min on ice.
27. Add prechilled (− 80 °C) acetone to the proteome to reach a final concentration of 85% (v/v) to precipitate proteins. Place the sample at − 80 °C for 30 min to allow complete precipitation of proteins.
  **! CAUTION** Acetone is highly flammable and toxic; handle with caution.
28. Gently centrifuge at 800×*g* at 4 °C for 5 min.
  ▲**CRITICAL** Do not exceed 800×*g* during centrifugation to avoid tight packing of the protein pellet, which can cause inefficient washing in the following steps.

**? TROUBLESHOOTING**

29. Resuspend the pellets in 5 mL of 85% (v/v) acetone on a vortex mixer for 2 min.
30. Centrifuge at 800×*g* at 4 °C for 5 min, collect the pellet, and discard the supernatant.
31. Repeat **steps 29–30** five to 10 times.
32. Vacuum- or air-dry the protein pellet.

■**PAUSE POINT** The dried pellet can be stored at − 80 °C until further processing.

#### Release Amide-Bonded Modifiers from the Denatured Proteome with CobB. ●TIMING: 4–5 H

33. Dissolve 0.5–2 mg protein from **step 32** in 1 mL deionized water, and sonicate for 5 min at room temperature to increase solubility.
  **?TROUBLESHOOTING**
34. Determine the protein concentration of the suspension using Bradford reagent.
35. Adjust the protein concentration of the suspension to 0.2 mg/mL.
36. To 1 mL proteome suspension, add recombinant CobB (purity > 98%; for reaction system, see Table 3; for preparation, see Table 4) to reach a proteome:CobB ratio of 100:1.
37. Allow the reaction to proceed at 37 °C for 4 h, and stop the reaction by adding 4 volumes of stop solution supplemented with 100 μM adonitol, which serves as an internal control for metabolite quantification in subsequent processes.
38. Store the stopped reaction at − 80 °C for more than 12 h to allow complete precipitation of all proteins in the reaction mix.
39. Centrifuge at 12,000×*g* for 10 min at 4 °C, collect the supernatant, and discard the pellet.
40. Vacuum dry the supernatant (which contains CobB-cleaved 2′-O-metabolite-ADP-ribose derivatives) in an Eppendorf vacuum drier at room temperature.
  **▲CRITICAL** Make sure the samples have been dried completely. Glass tubes should be capped immediately to avoid absorption of moisture from the environment.
41. Carry out LC/MS (go to **step 43**) and/or GC/MS (go to **step 46**) for analysis for metabolites.

**Table 3:** Reaction system for demodification by CobB TIMING: 0.5 h

The demodification reactions were carried out in a 600 μl reaction mix contains:	
Reagent name	Amount
pH 7.5 HEPES buffer (500 mM)	60 μl
DTT (100 mM)	6 μl
MgCl_2_ (600 mM)	6 μl
NAD^+^ (100 mM)	6 μl
Substrate proteome (10 μg/ml)	300 μl
CobB (5 mg/ml)	50 μl
PMSF (100 mM)	0.3 μl
ddH_2_O	Up to 600 μl

**Table 4:** Preparation of recombinant CobB TIMING: 2 days

1. Clone the *Salmonella enterica* deacetylase CobB gene (Supplementary Information 1 and 2) into the plasmid pET22b(+)-His.	
2. Introduce the constructed expression plasmid into *E. coli* BL21 (DE3) cells with standard CaCl_2_ transformation.	
3. Grown the transformed cells at 37 °C in LB medium containing 50 μg/mL ampicillin.	
4. Add IPTG to a final concentration of 1.0 mM when the optical density at 600 nm of the culture reaches 0.6–0.8.	
5. Keep the IPTG-induced cells growing for 6 h. Then, harvest the cells by centrifugation at 800×*g* for 5 min.	
■PAUSE POINT The harvested cells can be stored at − 20 °C for weeks before further processing.	
6. Disrupt the cells using an ultra-high pressure continuous flow cell disrupter in ice-cold 1× PBS containing 1 mM PMSF and 0.5 mM DTT.	
! CAUTION PMSF is highly toxic; handle with caution.	
7. Remove cell debris by centrifugation at 21,000×*g* for 40 min at 4 °C.	
8. Load the supernatant (10 mL) onto a Nickel resin column, and process the column with the *AKTA™ FPLC™* System.	
9. Wash the bound protein with 10 volumes of binding buffer, and elute the proteins using elution buffer.	
10. Desalt the proteins using gel chromatography with desalination buffer.	
11. Check the purity of the proteins using SDS-PAGE before storing at − 80 °C.	

#### LC/MS Analysis of Cleaved Metabolites ●TIMING: 2–3 H per Sample

42. Add 100 μL HCl (3 M in 1-butanol), and incubate for 15 min at 60 °C to release metabolites from the 2′-O-metabolite-ADP-ribose derivatives and form butyl esters.
43. Completely dry the samples under nitrogen, and refill with 100 μL injection solvent (80% water, 20% methanol, 0.1% formic acid).
44. Carry out LC/MS analysis directly using the prepared samples (for analysis settings, see Table 5).

**Table 5:** LC/MS settings

1. HPLC elution solvents	
The anionic ion-pair reagent HFBA was added to the mobile phase to improve analyte interactions with the stationary phase. An aqueous solution of HFBA (approximately 0.5 M) was diluted in water (mobile phase A) and methanol (mobile phase B) to a final concentration of 0.5 mM.	
2. Gradient system (pair of two alternating columns)	
The gradient system used two identical columns (Agilent Zorbax SB-C18; 2.1 mm × 50 mm, 1.8 μm; Waldbronn, Germany) connected to a 10-port two-position switching valve. Using the column switching valve and a second binary pump, the two columns were applied alternately. When one column was used for the analytical gradient, the other column was cleaned and re-equilibrated. From the prepared samples, 5 μL was injected into the HPLC system. The analytical gradient (pump 1; flow rate: 0.5 mL/min) increased linearly from 10% B to 50% B within 11 min. After isocratic elution for 0.5 min, the gradient returned to starting conditions until 11.6 min, and isocratic flow was performed for 10% B from 11.6 to 12.5 min at 0.5 mL/min. This short return of pump 1 to starting conditions was necessary for flushing the tubing from the pump to switching valve before the freshly equilibrated second column was switched in for the next injection. Parallel to the analytical gradient, the other column was cleaned and equilibrated by pump 2; within 1.0 min, the gradient was increased from 10% B to 95% B. The isocratic flow of 95% B was held for 4.5 min. From 5.5 to 6.5 min, initial conditions of the analytical gradient (10% B) were achieved and retained at isocratic flow until 12.5 min for flushing and equilibrating the column. Total time from injection to injection was 13.3 min (including the autosampler operation time of 0.8 min).	

**? TROUBLESHOOTING**

#### GC/MS Analysis of Cleaved Metabolites ●TIMING: 4–5 H per Sample

**▲CRITICAL** Samples subjected to GC/MS analysis need to be derivatized (●**TIMING 3 h)** before analysis.

45. Add 50 μL pyridine dissolved 250 mM methoxyamine hydrochloride to the samples from **step 40** to release metabolites from the 2′-O-metabolite-ADP-ribose derivatives (for reaction details, see reference 13).
  **! CAUTION** Methoxamine hydrochloride and pyridine are highly toxic; handle with caution. The derivation steps should be performed in a fume hood.
46. Incubate the reaction at 70 °C for 90 min with shaking.
47. To 50 μL solution from **step 41**, add MTBSTFA solution (100 μL, 20% in pyridine).
  **! CAUTION** MTBSTFA is highly toxic; handle with care.
48. Incubate the reaction at 30 °C for 60 min with shaking.

**▲CRITICAL** To avoid degradation by both MSTFA and the derivatized metabolites, strictly avoid moisture during manipulation.

49. Filter the derivatized products through a 0.22-μm syringe-driven filter unit. The derivatized metabolites are in the pass-through solution.
50. Transfer 100 μL derivatized metabolite solution into GC vials for GC/MS analysis (for settings, see Table 6).
  **▲CRITICAL** Analyze derivatized metabolites as soon as possible. The stability of derivatized metabolites at − 80 °C can range from a few days to a few months.

**Table 6:** GC/MS settings

▲CRITICAL Prior to GC-MS analysis, ensure that the instrument has been optimized. Details for our GC/MS workflow are listed below. Please be aware that other GC/MS methods with sufficient sensitivity, reproducibility, and linearity for the metabolites of interest can be used.	
1. Connect an Agilent 5977B Mass Spectrometer to an Agilent 7890B gas chromatograph. Use 5977MS/Enhanced MassHunter software to control the GC/MS system.	
GC settings:	
Column: Agilent 19091S-433HP-5MS 5% Phenyl Methyl Silox 325 °C: 29.8 m × 250 μm × 0.25 μm	
Carrier gas: helium (> 99.999% purity)	
Carrier gas flow rate: 1 mL/min	
Oven temperature program: 100 °C for 3 min, increase the temperature by 10 °C/min to 140 °C, increase the temperature by 8 °C/min to 260 °C, increase the temperature by 10 °C/min to 310 °C, and hold at this temperature for 5 min.	
Injection port temperature: 280 °C	
Transfer line temperature: 250 °C	
Injection volume: 1 μL	
Mode: splitless	
MS settings:	
Ionization mode: electron ionization (EI) mode	
Electron energy: 70 eV	
Ion source temperature: 250 °C	
Scan frequency: 2.7/s	
Mass range: 50.00–600.00 *m/z*	
2. Place the samples in the auto sampler tray for GC/MS analysis.	
3. Create a sample list (or sequence list) that includes the metabolomic sample details.	
4. Inject the pyridine standard at the beginning and the end of the sample analysis sequence.	

**?TROUBLESHOOTING.**

#### Data Analysis ●TIMING: 1–3 H for each Sample

51. Export the GC or LC peaks of each sample from **step 43** or **step 48** as a csv table.
52. Normalize peak areas in different tables to the internal control adonitol area.
53. Compare GC or LC peak areas of CobB-treated samples with those of matched untreated samples; those with 3-fold or higher increases represent possible positive hits.

**?TROUBLESHOOTING**

54. Obtain MS/MS spectra of possible positive hits and search against NIST mass spectral library to identify corresponding metabolites.

#### Validation of Positive Hits ●TIMING: 1–3 H for each Sample

55. Synthesize a peptide containing the desired modification on ε amine of lysine in the peptide.
56. Perform **steps 26–51**, check whether the positive identification of corresponding GC or LC peaks was increased by CobB, and produce MS/MS spectra with the synthetic modification.
57. Obtain authentic standard metabolites.
58. Generate GC or LC peaks and MS/MS spectra for the standard metabolite.
59. Compare the retention times and MS/MS spectra with those generated from experiment.
  **▲CRITICAL** Empirically, no more than a 0.5% retention time shift in GC or LC is tolerated.

**Troubleshooting**

**Table:** 

Step	Problem	Possible reason	solution
28	Tight pellet forming	High centrifugation speed or sticky pellet	Vortex to disperse pellet before further washing
33	Mild turbidity	Incomplete dissolving of denatured proteins	Centrifuge to remove
45	Unsteady ion response	Ion source parameters not optimized; unstable liquid flow	Optimize ion source parameters; check UPLC and connected systems for leaks
45	High noise levels (chemical/electronic)	Detector damaged and producing discharges	Arrange engineer visit
45, 50	Poor chromatographic peak shape	Column degradation or contamination	Replace the GC/UPLC column
45, 50	Carryover	The sample is too concentrated	Optimize injection volume and concentrate components in sample
45, 50	High mass spectrometer pressure	Gas infusion room atmosphere	Work through connections on MS for gas-tight seals; arrange engineer visit
50	Unstable GC gas flow or pressure	Leak in GC system	Work through connections on GC to check for gas-tight seals
50	Unsteady baseline	Detector or electronics fault	Arrange engineer visit
50	Absence or low number of peaks in all samples	Incomplete vacuum drying in step 40; failed injection	Ensure that the samples have been completely dried in step 40; re-inject the sample
53	Low number of positive hits	Low purity and activity of CobB	Preparation of recombinant CobB with high purity and activity
53	High amino acid intensity detected in the untreated samples (cell lysates alone/CobB alone)	Incomplete removing of non-covalently bonded small molecules from cell proteome/CobB	Repeat steps 29–30 more times; preparation of recombinant CobB with high purity

## Data Availability

Data of interest will be made available on request.

## Abbreviations

DTT: Dithiothreitol
EDTA: Ethylenediaminetetraacetic acid
GC/MS: Gas chromatography/mass spectrometry
IPTG: Isopropyl β-D-1-thiogalactopyranoside
LC/MS: Liquid chromatography/mass spectrometry
MTBSTFA: N-tert-Butyldimethylsilyl-N-methyltrifluoro-acetamide
PTM: Post-translational modification
SDS: Sodium dodecyl sulfate sodium salt
